# Supplementary figures and images for: Integrating population genetic structure, microbiome, and pathogens presence data in Dermacentor variabilis
Source: PeerJ. 2020 Jul 7;8:e9367. doi: 10.7717/peerj.9367 (PMC7350919; doi:10.7717/peerj.9367)

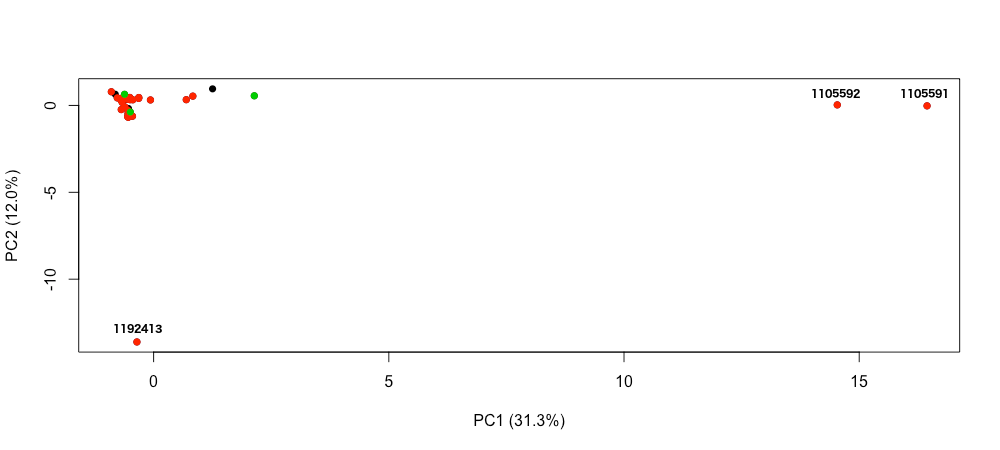

Supplement: Supplemental Information 2 — Axis 1 corresponds to the first principal component (PC) which explains 31.3% of the variance; and Axis 2 corresponds to the second PC, explaining an additional 12% of the variance. Samples are color-coded according to the genetic cluster they belong to: Eastern (red), Northern (black), and Western (green). The three samples that appear separated from the main cluster are labeled with the sample ID. [file peerj-08-9367-s002.tiff]

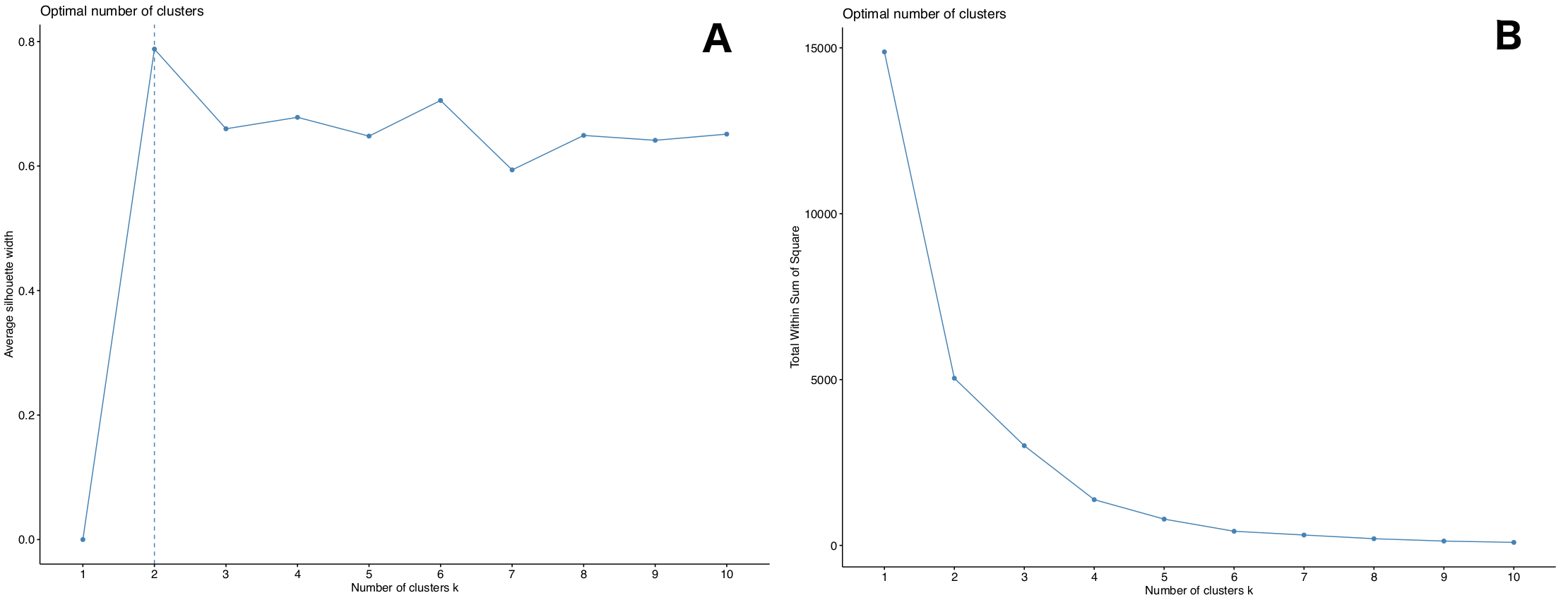

Supplement: Supplemental Information 3 — (A) The silhouette method shows k =2 as the optimal number of clusters; (B) the elbow method suggests k =4 as the ideal k. [file peerj-08-9367-s003.tiff]
